# Assessing PD-L1 Expression Level via Preoperative MRI in HCC Based on Integrating Deep Learning and Radiomics Features

**DOI:** 10.3390/diagnostics11101875

**Published:** 2021-10-12

**Authors:** Yuchi Tian, Temitope Emmanuel Komolafe, Jian Zheng, Guofeng Zhou, Tao Chen, Bo Zhou, Xiaodong Yang

**Affiliations:** 1 Academy of Engineering and Technology, Fudan University, Shanghai 200433, China; 18110860026@fudan.edu.cn; 2 School of Biomedical Engineering, Shanghai Tech. University, Shanghai 201210, China; tekomolafe@shanghaitech.edu.cn; 3 Department of Medical Imaging, Suzhou Institute of Biomedical Engineering and Technology, Chinese Academy of Sciences, Suzhou 215163, China; zhengj@sibet.ac.cn; 4 Department of Radiology, Zhongshan Hospital, Shanghai 200032, China; zhou.guofeng@zs-hospital.sh.cn; 5 School of Information Science and Technology, Fudan University, Shanghai 200433, China; eetchen@fudan.edu.cn; 6 Department of Interventional Radiology, Zhongshan Hospital, Shanghai 200032, China; 7 National Clinical Research Center for Interventional Medicine, Shanghai 200032, China

**Keywords:** radiomics, deep learning, hepatocellular carcinoma, PD-L1, immunotherapy

## Abstract

To assess if quantitative integrated deep learning and radiomics features can predict the PD-L1 expression level in preoperative MRI of hepatocellular carcinoma (HCC) patients. The data in this study consist of 103 hepatocellular carcinoma patients who received immunotherapy in a single center. These patients were divided into a high PD-L1 expression group (30 patients) and a low PD-L1 expression group (73 patients). Both radiomics and deep learning features were extracted from their MRI sequence of T2-WI, which were merged into an integrative feature space for machine learning for the prediction of PD-L1 expression. The five-fold cross-validation was adopted to validate the performance of the model, while the AUC was used to assess the predictive ability of the model. Based on the five-fold cross-validation, the integrated model achieved the best prediction performance, with an AUC score of 0.897 ± 0.084, followed by the deep learning-based model with an AUC of 0.852 ± 0.043 then the radiomics-based model with AUC of 0.794 ± 0.035. The feature set integrating radiomics and deep learning features is more effective in predicting PD-L1 expression level than only one feature type. The integrated model can achieve fast and accurate prediction of PD-L1 expression status in preoperative MRI of HCC patients.

## 1. Introduction

Hepatocellular carcinoma (HCC) is the third leading cause of cancer-related death worldwide [1]. It is the second and sixth most common type of cancer in men and women, respectively, worldwide. Annually, there are approximately 905,677 new cases and 830,180 deaths caused globally [2]. Currently, immunotherapy by immune checkpoint inhibitor (ICI) has emerged as a crucial therapeutic option for improving the prognosis of various cancers [3,4,5,6,7,8]. This has demonstrated promising efficacy of treatment over conventional chemotherapy for different malignant tumors [9,10,11,12,13,14], including HCC [15,16], by triggering the antitumor immune response of T cells instead of directly targeting the tumor itself [17]. However, the rates of objective response to immune checkpoint inhibitors vary from person to person. Only 20–50% of patients respond to immune checkpoint inhibitors [18]. Therefore, it is necessary to preoperatively select the ideal HCC for quick and accurate immunotherapies in patients, which can improve the efficacy of treatment and the overall survival rate. Previous studies have shown that the expression status of programmed death-ligand 1 (PD-L1) in tumors is related to the clinical outcome and treatment response of PD-L1 pathway inhibition [19,20,21,22,23,24,25], and it can be used as a predictive biomarker for ICI therapy [26,27,28]. Generally, cancer patients are sensitive to immunotherapy when PD-L1 expression level exceeds 50% [29,30,31]. Therefore, preoperatively analyzing the PD-L1 expression level can assist doctors to identify which patients are suitable for ICI therapy [32]. At present, the examination methods of invasive biopsy immunohistochemistry (IHC) and genomics evaluation are the conventional method of detecting PD-L1 expression status [33,34,35]. However, these methods are limited in clinical application due to invasiveness, unrepeatability, and time consumption. Sometimes, it is also not easy to obtain tissue specimens from inaccessible locations. Therefore, clinical practice is urgently in need of a quick, reliable, and noninvasive method for assessing PD-L1 expression.

Radiomics, a high-dimensional quantitative feature analysis approach, can extract high-throughput features from medical imaging and perform quantitative analysis of tumor heterogeneity. Several studies have shown that radiomics features could characterize the tumor and its tumor microenvironment (TME) [36,37,38,39,40,41,42], and were closely related to specific microscopic features at genes, proteins, and molecular levels. Radiomic features have been suggested in the application of predicting molecular subtyping, tumor gene expression, pathological classification, treatment response, and survival [38,39,40,41,42,43,44]. Deep learning is a class of machine learning techniques that can extract a large number of higher-level deep features from deep hidden layers of convolution neural network (CNN), which has been widely adopted in image recognition and image classification. Compared with handcrafted features, these deep features contain more abstract medical image information and provide more predictive patterns. However, the main drawbacks of deep learning are the requirement of massive data for training classifiers and the poor interpretability of features, which limits its application. Recently, there have been many studies using radiomics or deep learning to predict the PD-L1 expression level in HCC patients [45,46,47,48,49,50,51]. All these studies gave good exploration for predicting the expression level of PD-L1, but there are still relatively few studies exploiting deep learning and radiomics features for operatively assessing the PD-L1 expression status. They only applied the statistical feature from radiomics or deep learning to qualitatively analyze the expression level of PD-L1, but lacking the quantitative analysis of specific characteristics relating to the expression.

In this study, we propose an integrated model for assessing PD-L1 expression level in HCC patients by integrating deep learning and radiomics features in preoperative MRI, and then carry out a quantitative analysis of the features. We only use the deep learning model as a feature extractor (not a classifier), and then integrate the deep learning features into the radiomics analysis model, which enriches the predictive power of the model and improves the overall performance of the model with limited training data. Our goal is to assess whether quantitative integrated deep learning and radiomics features can predict the PD-L1 expression level in preoperative MRI of HCC patients.

## 2. Materials and Methods

### 2.1. Patients

This is a retrospective single-center study, that consisted of 103 HCC patients who received treatment in our center between December 2017 and May 2021. The hospital’s ethical review committee approved the study. The inclusion criteria of cases enrolled were as follows: (1) patients with pathologically proven HCC; (2) abdominal MRI performed within one month before surgery; (3) availability of tumor samples and clinical data; (4) IHC examination of PD-L1 performed. Exclusion criteria were (1) the interval between MRI examination and surgery was more than 1 month; (2) poor MR imaging quality; (3) patients with other tumor diseases than HCC at the same time.

### 2.2. Detection of PD-L1 Expression Status

The PD-L1 expression level was measured by the IHC test with a detection kit of Ventana PD-L1 (SP142). The test specimen was paraffin tumor tissue obtained by surgical intervention. The specimen was fixed by a concentration of 10% neutral formalin for 6–8 h, regarding full circumferential or partial cell membrane staining as PD-L1 positive tumor cells. The PD-L1 high expression was defined as the percentage of PD-L1-positive tumor cells relative to the total tumor cells exceeding 50%; otherwise, it was defined as PD-L1 low expression. Finally, the 103 enrolled patients were divided into two groups by PD-L1 expression level: a high PD-L1 expression group (30 patients) and a low PD-L1 expression group (73 patients).

### 2.3. Image Acquisition and Tumor Segmentation

**Figure 1:**
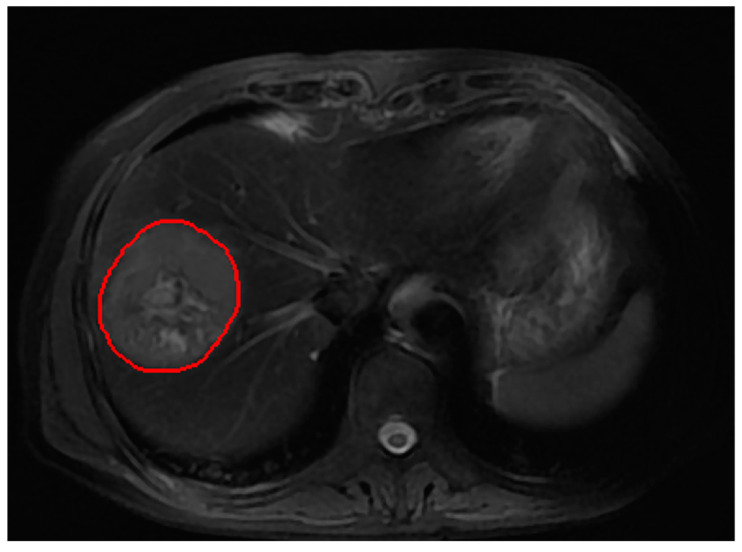
An example of the tumor VOI segmentation on T2WI.

All MR images were acquired on a 3.0T MRI scanner device (Verio; Siemens, Erlangen, Germany). The MRI protocol consisted of T2 weighted TSE sequence (TR/TE = 5632 ms/120 ms; acquired resolution of 0.74 mm × 0.74 mm, slice thickness = 6 mm; matrix = 512 × 512). The segmentation of the entire tumor volume of interests (VOI) was manually performed slice by slice on T2-WI by a radiologist with 10 years of experience; after that, each segmentation slice was reviewed and modified by a chief radiologist, who had over 20 years of experience in MRI. The region of interest (ROI) covered the whole tumor. The Medical Imaging Interaction Toolkit (MITK) v2018.04 software was applied to draw the tumor VOI. Figure 1 shows an example of the tumor VOI in a sequence. The size of each MR image was adjusted to 256 × 256, and the intensity value on T2-WI was normalized by N4BiasFieldCorrection [52] and the intensity range was standardized using histogram matching [53].

### 2.4. Feature Extraction

#### 2.4.1. Radiomics Features (RsF)

A total of 1595 3-D radiological features were extracted from each VOI with Pyradiomics [54]. These radiomic features could be divided into three categories: texture characteristics, intensity characteristics, and geometry characteristics. The texture characteristics of VOI could be described by 16 gray-level size zone matrix (GLSZM) features, 24 gray-level co-occurrence matrix (GLCM) features, 5 neighboring gray-tone difference matrix (NGTDM) features, 16 gray-level run-length matrix (GLRLM) features, and 14 gray-level dependence matrix (GLDM) features. The GLSZM can quantify gray level zones in an image, which preserves the size and number of connected domains of all grayscales in the image; the GLCM calculates how often different combinations of gray levels co-occur in an image, which reflects the variation in intensity at the pixel; the NGTDM quantifies the difference between a gray value and the average gray value of its neighbors within a certain distance; the GLRLM quantifies gray level runs, which creates statistics and records the distribution and relationship of image pixels; the GLDM quantifies gray level dependencies in an image, which calculates the number of connected voxels within a certain distance that is dependent on the center voxel. In medical image analysis, texture features are widely used to quantitatively describe the characteristics of lesions. The intensity characteristics within the tumor were reflected by 18 first-order statistical features. The geometry characteristics of the tumor were described by 14 three-dimensional shape features. In addition, eight kinds of image filters were also applied to the original image, respectively, to yield its corresponding derived image. The filters included gradient, wavelet, square, square root, logarithm, exponential, Laplacian of Gaussian (LoG), and local binary pattern 3D (LBP-3D). The above radiomic features except shape features were also extracted from these derived images.

#### 2.4.2. Deep Learning Features (DLF)

A total number of 1024 deep learning features for each patient were extracted from a three-dimensional convolution neural network (3D-CNN), which consists of two 3D convolution layers and two fully connected layers. The deep features were obtained from the outputs of the first fully connected layer after applying the rectified linear unit (ReLU) [55] activation function, which changed values to be 0 if the values <0. The architectures and parameters used are described in Table 1. The 3D-CNN architecture was designed with Tensorflow [56]. The training batch size was set as 20, and the model was trained by Adam [57] optimizer with a learning rate of 10^−3^ and 50 epochs.

#### 2.4.3. Integrated Features (RsF+DLF)

**Figure 2:**
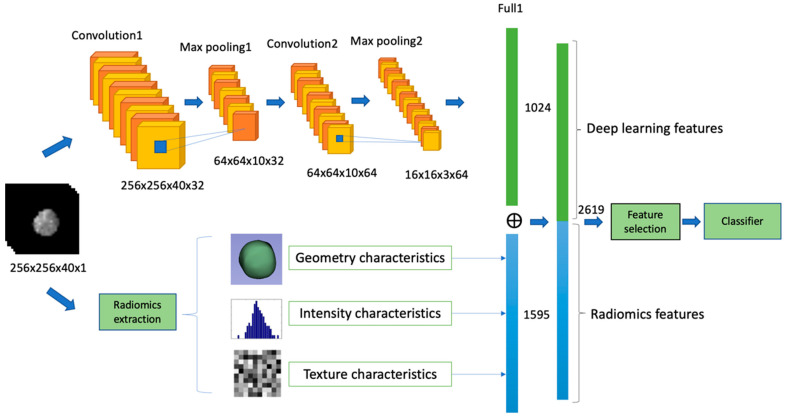
The predictive models from the integration of radiomics and deep features.

The output features of the first fully connected layer of the 3D-CNN framework were connected with the latter half of the radiomics process so that the extracted 1595 radiomics features and 1024 deep learning features were merged into an integrative feature space. The total number of features after fusion was 2619, as shown in Figure 2. After feature reduction, then fed into the machine learning classifier for training.

### 2.5. Feature Selection and Classifier Modeling

**Figure 3:**
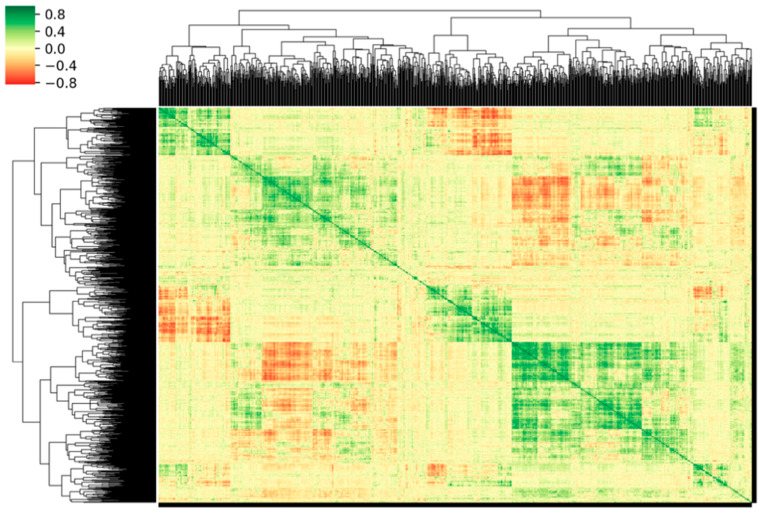
Cluster gram of significant correlations, expressed as the Pearson correlation coefficient, between the candidate features. Correlation degrees are colored according to the color bar shown on the left.

We applied normalization to the feature matrix. Each feature vector was subtracted from the average value of the vector and then divided by its length. Due to the high dimensionality of the feature space, we utilized the Pearson correlation coefficient (PCC) [58] analysis to identify redundant features. One of the feature pairs would be removed when the PCC absolute value was higher than 0.86, which is considered to be redundant. The correlation matrix of the candidate features was calculated and depicted as a correlation cluster gram as shown in Figure 3. The deeper color indicates the stronger correlation. After that, the dimensionality of the feature space was reduced and each feature was independent of the others. Before building the classifier model, a recursive feature selection approach, Support Vector Machine-Recursive Feature Elimination (SVM-RFE) was used to select features. The SVM-RFE method has been proven to be very effective in finding a worthwhile and significant feature for improving classification performance [59,60], which selects features based on the SVM classifier by recursively considering the smaller size of feature sets. The SVM-RFE algorithm obtained a ranking list of all features by eliminating only one feature that had the least impact on the prediction of the SVM model each time [61,62]. The first item in the ranking list was the most relevant feature, while the last item had the least relevant feature. Finally, the ranking list of the top-ranked features was selected to build the SVM model. Here we used a linear kernel function for building the SVM classifier model [63] with these selected features, which could make it easier to interpret the characteristic coefficients of the final model [64].

### 2.6. Statistics

The univariate analysis method was used to evaluate the statistical significance of patients’ clinical characteristics in the prediction of PD-L1 expression. A two-sided *p*-value < 0.05 was considered significant and *p*-value < 0.01 was extremely significant. The ROC curve was used to assess the predictive ability of the model, and its corresponding quantification was calculated by the AUC. The accuracy, recall, specificity, precision, and f1_score were also calculated at the cutoff value that maximized the AUC value [65]. To validate the performance of the model, we adopted 5-fold cross-validation on the dataset. In the scenario of 5-fold cross-validation, the dataset was randomly divided into five unique subsets S = [s1, s2, s3, s4, s5] to train five independent models. The first model was trained using the subsets [s2, s3, s4, s5] and tested using s1, while the second model was trained using [s1, s3, s4, s5] and tested using s2. This procedure was repeated until all five subsets had been tested. We ensured that no patient was overlapping between the subsets. The mean and standard deviation of the above-mentioned metrics were calculated to evaluate the overall model performance. In our case, the amount of patients is 103 (high: 30; low: 73), so in each fold cross-validation, the test set includes about 20 patients and the training set contains roughly 80 patients. There is no extra validation set, duo to the limited number of patients. The Keras framework was used to conduct feature selection, feature extraction, classifier modeling, and statistical analysis. The experiment code was implemented on an Nvidia GeForce GTX 1070 GPU with 8 GB of GDDR5 memory.

## 3. Results

### 3.1. Patient Clinical Characteristics

Among 103 patients, 30 patients (age median: 55.20 ± 11.05) with tumor cells expressing PD-L1 ≥ 50% were assigned into the high expression group, and 73 patients (age median: 54.48 ± 12.70) with expression scored < 50% were assigned to the low expression group. In our univariate analysis, there was no significant difference in clinical characteristics including Age, Sex, HBV_DNA, HBs, AFP, Maximal tumor diameter, CEA, and TBIL between the two PD-L1 expression groups (*p* > 0.05 for all) (Table 2).

### 3.2. Comparison of Different Classifiers

The experiment compared the classification performance of different classifiers, and the best-performing classifier was used to further verify the performance of strategies based on different features using five-fold cross-validation. The compared classifiers are SVM, AE, LR-Lass, Decision Tree, Random Forest, and LDA. All classifiers were trained (high: 20; low: 53) and tested (high: 10; low: 20) on the same dataset. The predictive results are shown in Table 3 below. According to the predictive indicators, the overall performance of the SVM classifier is better than other classifiers with the highest AUC score of 0.758. Therefore, SVM was selected as the best classifier for further verification experiments.

### 3.3. Feature Selection and Signature Building

The integrated predictive model with the best AUC is based on 14 selected features, and the details are shown in Table 4. Through SVM with linear kernel function, the 14 features were weighted by their respective coefficients, the integrated score for each patient can be computed as follows:

RsF+DLF_Score = (−0.995) × original_glcm_InverseVariance + (0.742) × wavelet_HHL_firstorder_Mean + (−0.748) × wavelet_HHL_glcm_InverseVariance + (−0.714) × gradient_ngtdm_Contrast + (0.819) × squareroot_glcm_ClusterTendency + (1.116) × deep_feature_81 + (1.005) × deep_feature_193 + (−0.841) × deep_feature_486 + (−1.064) × deep_feature_524 + (−0.953) × deep_feature_629 + (1.142) × deep_feature_670 + (1.240) × deep_feature_805 + (1.142) × deep_feature_841 + (0.813) × deep_feature_889.

### 3.4. Prediction for PD-L1 Expression Level

**Figure 4:**
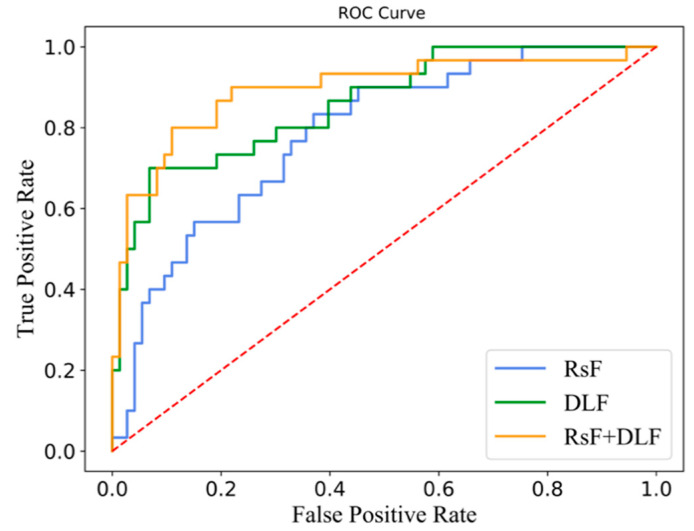
ROC curves of the prediction models.

Table 5 shows the predictive performance of the radiomics-based model, deep learning-based model, and integrated model on five-fold cross-validation. The integrated model achieved the best ability of prediction, with a value of AUC 0.897 ± 0.084, accuracy 0.887 ± 0.041, f1-score 0.764 ± 0.106, specificity 0.981 ± 0.029, precision 0.948 ± 0.076, and recall 0.660 ± 0.167, respectively, followed by the deep learning-based model with an AUC of 0.852 ± 0.043, and finally radiomics-based model with AUC 0.794 ± 0.035. The ROC curves for the three models are shown in Figure 4. The blue line represents the radiomics feature-based model; the green line corresponds to the deep learning feature-based model; the orange line represents the model with radiomics and deep learning features with the best result.

## 4. Discussion

Immunotherapy is gradually becoming the main therapy option for HCC treatment in the clinic. Preoperatively assessing the immune status of patients can assist the physicians to identify which patients are suitable for immunotherapy, thereby improving treatment efficiency and overall survival rate. The expression level of PD-L1 plays an important role in guiding the selection of ICI therapy for liver cancer patients. The utilization of radiomics features for preoperatively assessing PD-L1 expression before ICI therapy has attracted significant attention in the literature [46,47,48,49,50,51]. For example, Hectors et al. [46] combined quantitative and qualitative radiomics features to preoperatively assess the expression of PD-L1 biomarker in resected HCC. They identified significant associations of texture characteristics and the ratios of quantitative enhancement with PD-L1 expression. Zhang et al. [48] converted the MRI radiomic features of liver tumors into a quantitative Radscore for preoperatively predicting the expression of PD-L1, achieving an AUC of 0.750. They found that the radiomics features related to PD-L1 expression largely originated from GLCM features. Liao et al. [51] utilized the CT radiomics features from GLRLM and GLCM in HCC patients to build the predictive model for PD-L1 expression. These studies demonstrated that the analysis method of radiomics could be used to predict PD-L1 expression status, which might aid in the stratification of liver cancer patients for ICI. However, they only exploited radiomics features for operatively assessing the PD-L1 expression status, and did not incorporate the features from deep learning. 

Although the features of deep learning are poorly interpretable, it tends to improve the prediction performance by integrating radiomics and deep learning features. However, there are still relatively few studies exploiting deep learning and radiomics features for operatively assessing the PD-L1 expression status. In this study, we presented an integrated model for quick and accurate assessment of PD-L1 expression level in HCC patients before immunotherapy, our method is based on the analysis of MRI radiomics features and deep learning features together. In addition, we carried out a quantitative analysis of these features, and the significant relationship of radiomics features and deep learning features with PD-L1 expression of IHC. As assessed by the five-fold cross-validation, the integrated model achieved the highest AUC of 0.897 ± 0.084, while the deep learning-based and radiomics-based model obtained the AUC of 0.852 ± 0.043 and 0.794 ± 0.035, respectively. The experimental results demonstrated that the feature set that integrates radiomics and deep learning features is more effective in predicting PD-L1 expression level than using only one feature type from preoperative MRI of HCC patients. Additionally, we found that our integrated model based on 14 features achieved the highest AUC, of which 5 and 9 were radiomics deep learning features, respectively. Both the deep learning features and radiomics features contributed to the prediction of PD-L1 expression status, but the deep learning features contributed more. We then formulated the predictive score equation according to these 14 features.

**Figure 5:**
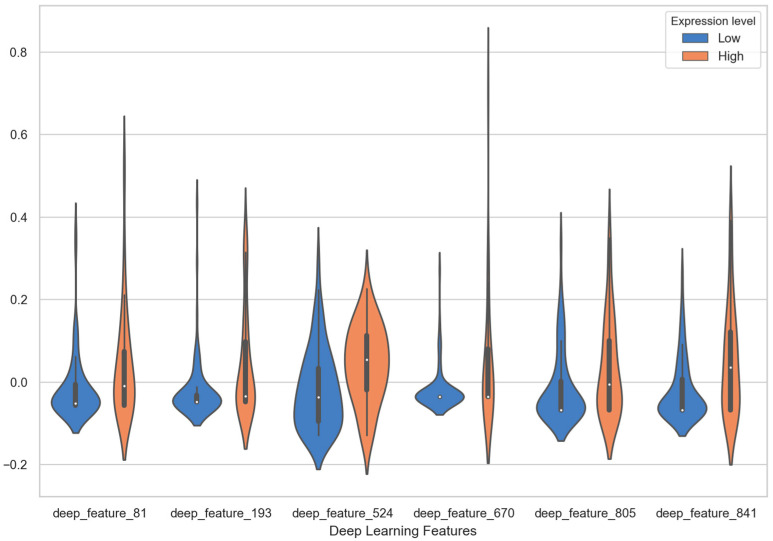
The violin plot of six deep learning features with high statistical significance distributions between the high PD-L1 expression level group and low expression level group.

**Figure 6:**
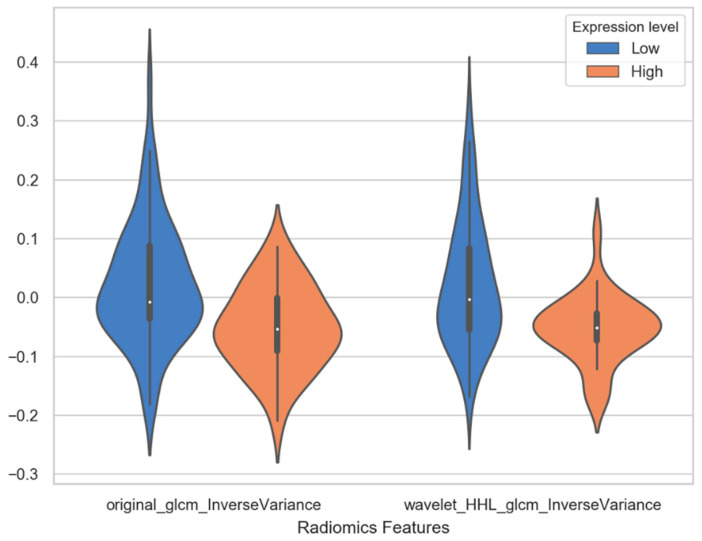
The violin plot of two radiomics features distributions between the high PD-L1 expression level group and low expression level group.

**Figure 7:**
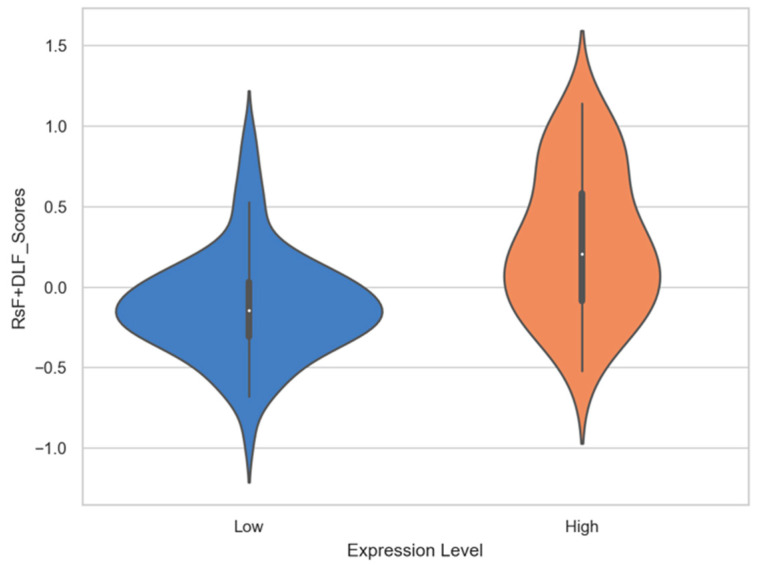
The violin plot of RsF+DLF_Scores distributions between the high PD-L1 expression level group and low expression level group.

Several MRI radiomics features and deep learning features had shown correlation with PD-L1 expression. In deep learning features, six deep learning features have statistical significance to expression level of PD-L1, which are deep_feature_524 (*p* = 0.003), deep_feature_841 (*p* = 0.006), deep_feature_193 (*p* = 0.012), deep_feature_670 (*p* = 0.019), deep_feature_81 (*p* = 0.028) and deep_feature_805 (*p* = 0.031). The results of statistical significance indicate that these characteristics are correlated with the expression level of PD-L1. The number in the deep_features represents the order of neurons in the fully connected layers. For example, deep_feature_524 indicates that this feature is taken from the 524th neuron of the fully connected layer. As shown in the column of coefficients in Table 4, the coefficients of six features in SVM linear function are all positive, which means that these feature values are positively correlated with the expression of PDL1 level. The violin plot of feature distributions between the high PD-L1 expression level group and low expression level group is shown in Figure 5. Almost all the median values of deep learning features in the high expression group are higher than the median of the low expression group. Especially the deep_feature_524 and deep_feature_841 with extremely statistical significance (*p*-value < 0.01); its scores in the high expression group are mainly distributed in the positive value area, while the group in the low expression group are distributed in the negative value area, and there is a significant difference in distribution between the two groups. Among the features of radiomics, original_glcm_InverseVariance and wavelet_HHL_glcm_InverseVariance are strongly related to PD-L1 expression prediction with *p* values of 0.001 and 0.001, respectively. In addition, their coefficients in SVM linear function are all negative, with value of −0.995 and −0.748 (in Table 4). It indicates the two features are negatively correlated with the expression of PDL1 level. A significant difference is observed between original _glcm_InverseVariance values of the PD-L1 high expression and low expression group (Figure 6, left panel), whose median in the high group is lower than the low group. For the wavelet_HHL_glcm__InverseVariance feature, the high group also has a lower median value than the low group in Figure 6 (right panel). The GLCM feature can characterize the texture of an image; it can reflect the distribution of co-occurring pixel values at a given offset, by calculating the frequency of pixel pairs with specific values and specific spatial relationships in the image. It has been used as an approach to texture analysis in medical image analysis [66,67]. This result demonstrated that the distribution of co-occurring pixel values in the tumor area may be related to the PD-L1 expression status and high expression level with a high GLCM score. Wavelet filters can make weak signals be recovered from noise, especially in the processing of magnetic resonance images. Wavelet filters are mainly used to optimize radiomics features, which can quantify the heterogeneity of tumors at different scales. Previous studies have demonstrated that wavelet features could be an important predictor for constructing radiomic features [68,69,70]. The RsF+DLF_Score shows an extremely statistical significance to PD-L1 expression status with *p* = 2.502 × 10^−9^. The score is higher in the high expression group (0.27 ± 0.44) than in the low expression group (−0.11 ± 0.33) as shown in Figure 7. This result demonstrated that there is an obvious difference in RsF+DLF_Score between the high PD-L1 expression group and the low expression group. The RsF+DLF_Score can be used to reflect the level of PDL1 expression, with the higher score value, the higher PD-L1 expression level. These findings of this study will provide helpful indications for the immunotherapy of HCC patients.

However, there are some limitations to our study. First, it is a retrospective study from a single center without additional validation by other hospitals, and the number of HCC cases with the PD-L1 test was limited, especially the proportion of patients with high PD-L1 expression. Therefore, the validity of our research results might have been impaired. In future research, more cases and multi-center datasets are needed. Second, although we have given the features that contribute to predicting PD-L1 expression status in the combined model, only the features of radiomics are interpretable, while the interpretability of the deep learning features is still poor. The possible correlation between the tumor biological mechanisms and the deep learning features will be investigated from molecular, proteins, and gene levels in future work. Additionally, due to the data collection issue, only T2-WI sequence of MRI was available to be analyzed in this study, which may neglect information from other kinds of sequences.

## 5. Conclusions

We proposed a prediction model of integrating the features of radiomics and deep learning for quick and accurate assessment of PD-L1 expression levels for HCC patients before ICI therapy. Our integrated model achieves good performance on predicting PD-L1 expression level in preoperative MRI. These results suggest that MRI radiomics combined with deep learning analysis shows potential for clinical practice in the assessment of PD-L1 expression in HCC, which may achieve the stratification of HCC patients for immunotherapy.

## Figures and Tables

**Table 1:** 3D-CNN Architecture for extraction of deep learning features.

Layers	Parameter Setting
Conv 3D-1	size = 5 × 5 × 5; stride = 1; zero-padded
Relu-1	Alpha = 0.2
Max Pool 3D-1	size = 4 × 4 × 4; stride = 4; zero-padded
Conv 3D-2	size = 5 × 5 × 5; stride = 1; zero-padded
Relu-2	Alpha = 0.2
Max Pool 3D-2	size = 4 × 4 × 4; stride = 4; zero-padded
Fully connected-1	
Flat-1	
Relu-3	Alpha = 0.2
Dropout-1	*p* = 0.5
Fully connected-2	
SoftMax	

**Table 2:** Comparisons of clinicopathologic characteristics of HCC patients between PD-L1 high-expression group and low-expression group.

VariablesS	High-Expression (PD-L1 ≥ 50%)	Low-Expression (PD-L1 < 50%)	*p* Value
Age	55.20 ± 11.05	54.48 ± 12.70	0.077
Sex			0.369
Male	26 (86.7%)	58 (79.5%)	
Female	4 (13.3%)	15 (20.5%)	
HBV_DNA			0.224
Positive	13 (43.0%)	17 (23.3%)	
Negative	17 (57.0%)	56 (76.7%)	
HBs			0.182
Positive	25 (83.3%)	51 (69.9%)	
Negative	5 (16.7%)	22 (30.1%)	
AFP(ng/mL)			0.260
≤20	11 (36.7%)	24 (32.9%)	
>20	19 (63.3%)	49 (67.1%)	
Maximal tumor diameter			0.903
≤5	19 (63.3%)	41 (56.2%)	
>5	11 (36.7%)	32 (43.8%)	
CEA	2.61 ± 1.48	2.56 ± 1.53	0.427
TBIL	13.99 ± 5.28	13.28 ± 5.71	0.962

*p*-value is calculated by the univariate analysis between PD-L1 expression level and each of the clinicopathologic variables. Statistically significant level: significant (*p* value < 0.05); highly significant (*p* value < 0.01); HBV_DNA = Hepatitis B virus DNA; HBs = Hepatitis B surface AFP = alpha fetoprotein; CEA = carcinoembryonic antigen; TBIL = total bilirubin.

**Table 3:** Performance comparison of different classifiers.

Model	Accuracy	AUC	Negative Predictive	Positive Predictive	Sensitivity	Specificity
SVM	0.786	0.758	0.859	0.625	0.667	0.836
AE	0.708	0.677	0.794	0.500	0.500	0.794
LR-Lasso	0.553	0.675	0.885	0.382	0.866	0.424
Decision Tree	0.737	0.678	0.810	0.551	0.533	0.821
Random Forest	0.737	0.706	0.819	0.548	0.566	0.808
LDA	0.747	0.724	0.873	0.551	0.733	0.753

**Table 4:** Results of statistical analysis of integrated features selected.

Features	High-Expression	Low-Expression	Coefficient	*p* Value
original_glcm_InverseVariance	−0.05 ± 0.07	0.02 ± 0.10	−0.995	0.001
wavelet_HHL_firstorder_Mean	0.03 ± 0.12	−0.01 ± 0.08	0.742	0.286
wavelet_HHL_glcm_InverseVariance	−0.05 ± 0.06	0.02 ± 0.10	−0.748	0.001
gradient_ngtdm_Contrast	0.01 ± 0.07	0.00 ± 0.11	−0.714	0.064
squareroot_glcm_ClusterTendency	0.02 ± 0.12	−0.01 ± 0.09	0.819	0.234
deep_feature_81	0.04 ± 0.13	−0.02 ± 0.08	1.116	0.028
deep_feature_193	0.04 ± 0.13	−0.02 ± 0.08	1.005	0.012
deep_feature_486	−0.02 ± 0.10	0.01 ± 0.10	−0.841	0.310
deep_feature_524	0.04 ± 0.09	−0.02 ± 0.10	1.064	0.003
deep_feature_629	−0.02 ± 0.07	0.01 ± 0.11	−0.953	0.278
deep_feature_670	0.04 ± 0.16	−0.02 ± 0.05	1.461	0.019
deep_feature_805	0.03 ± 0.12	−0.01 ± 0.09	1.240	0.031
deep_feature_841	0.05 ± 0.13	−0.02 ± 0.07	1.142	0.006
deep_feature_889	0.03 ± 0.15	−0.01 ± 0.06	0.813	0.503
RsF+DLF _Score	0.27 ± 0.44	−0.11 ± 0.33	None	2.502 × 10^−9^

*p*-value is calculated by the Wilcoxon rank-sum test between PD-L1 expression level and each of the clinicopathologic variables. Statistically significant level: significant (*p* value < 0.05); highly significant (*p* value < 0.01).

**Table 5:** The performance of radiomics-based model, deep learning-based model and integrated model on 5-Fold cross validation.

Model	AUC	Accuracy	f1-Score	Specificity	Precision	Recall
RsF	0.794 ± 0.035	0.766 ± 0.094	0.494 ± 0.212	0.916 ± 0.077	0.687 ± 0.301	0.400 ± 0.190
DLF	0.852 ± 0.043	0.854 ± 0.050	0.703 ± 0.131	0.947 ± 0.087	0.892 ± 0.166	0.633 ± 0.217
RsF+DLF	0.897 ± 0.084	0.887 ± 0.041	0.764 ± 0.106	0.981 ± 0.029	0.948 ± 0.076	0.660 ± 0.167

## Data Availability

The data presented in the study are available on request from the corresponding author.
